# Three new species of *Tasmaniosoma* Verhoeff, 1936 (Diplopoda, Polydesmida, Dalodesmidae) from northeast Tasmania, Australia

**DOI:** 10.3897/zookeys.488.9460

**Published:** 2015-03-19

**Authors:** Robert Mesibov

**Affiliations:** 1 Queen Victoria Museum and Art Gallery, 2 Invermay Road, Launceston, Tasmania 7248, Australia

**Keywords:** Diplopoda, Polydesmida, Dalodesmidae, millipede, Australia, Tasmania

## Abstract

The small-range millipedes *Tasmaniosoma anubis* **sp. n.**, *Tasmaniosoma interfluminum* **sp. n.** and *Tasmaniosoma nicolaus* **sp. n.** are described, and the colour of live *Tasmaniosoma barbatulum* Mesibov, 2010 is documented.

## Introduction

When reviewing *Tasmaniosoma* Verhoeff, 1936 several years ago I wrote that “more small-range species may remain to be discovered” (Mesibov 2010: 32). Here I describe three new *Tasmaniosoma* from northeast Tasmania with known range envelopes of <12, <40 and ca 100 km^2^. *Tasmaniosoma* now contains 22 species, or about one quarter of the 86 species in the suborder Dalodesmidea so far described from Tasmania (Mesibov 2006–2015).

## Methods

**Figure 1.**
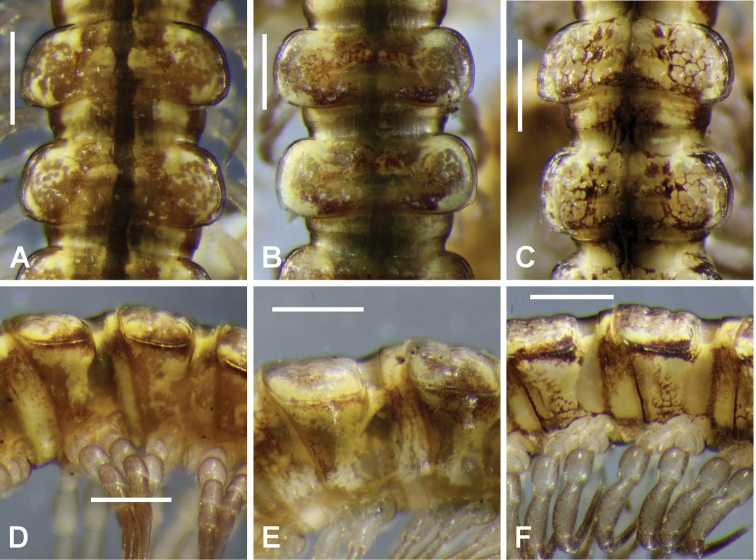
Dorsal **A–C** and right lateral **D–E** views of midbody rings of freshly killed males **A, D** *Tasmaniosoma anubis* sp. n., ex QVM 23:53865 **B, E** *Tasmaniosoma interfluminum* sp. n., ex QVM 23:53866 **C, F** *Tasmaniosoma nicolaus* sp. n., paratype ex QVM 23:53860. Scale bars = 0.5 mm.

**Figure 2.**
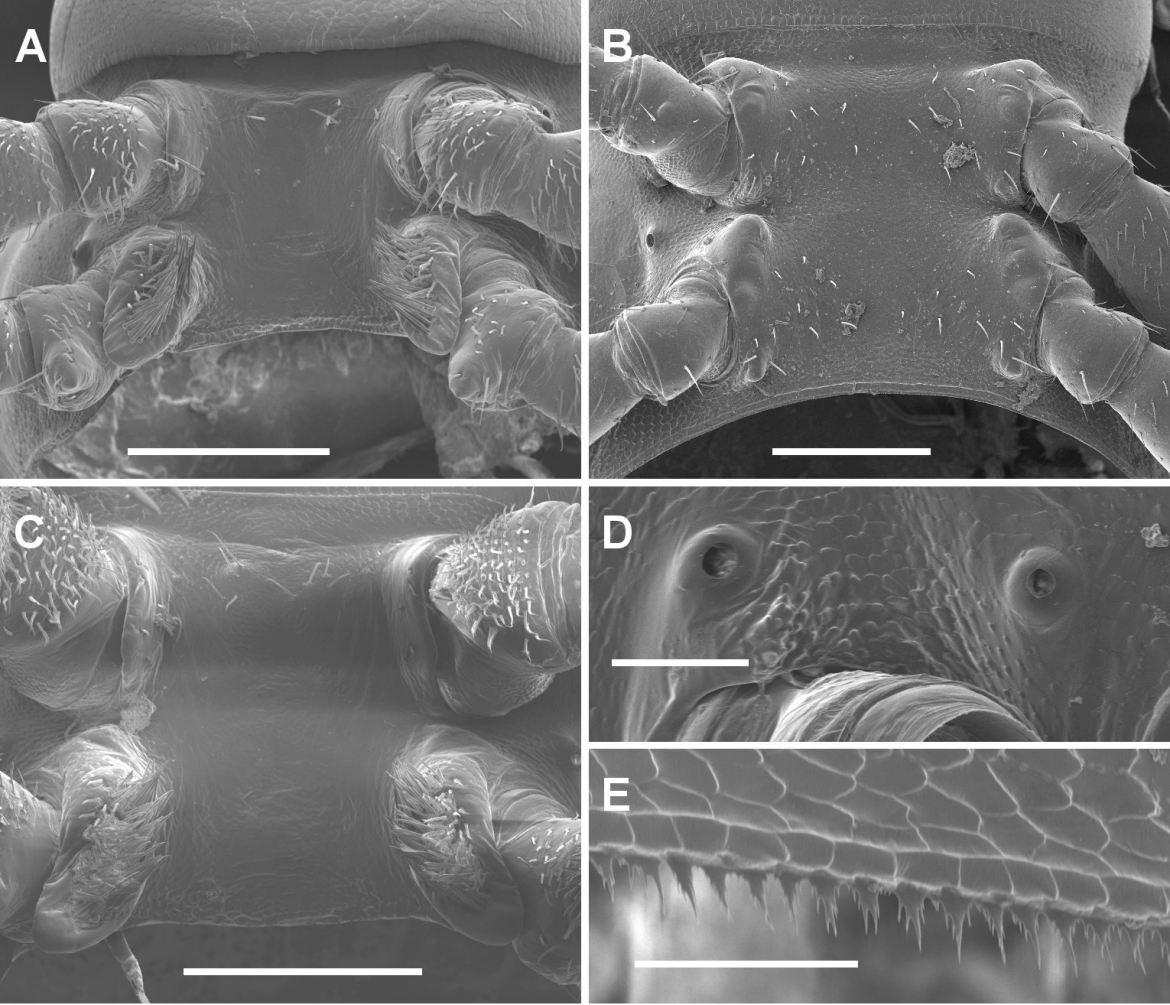
**A, D, E** *Tasmaniosoma anubis* sp. n., paratype ex QVM 23:53817 **B** *Tasmaniosoma interfluminum* sp. n., paratype ex QVM 23:52247 **C** *Tasmaniosoma nicolaus* sp. n., paratype ex QVM 23:53860 **A, B, C** Ventral views of male ring 6 **D** Left lateral view of midbody spiracles **E** Limbus on lateral portion of midbody ring. Scale bars: **A–C** = 0.25 mm, **D, E** = 0.05 mm.

**Figure 3.**
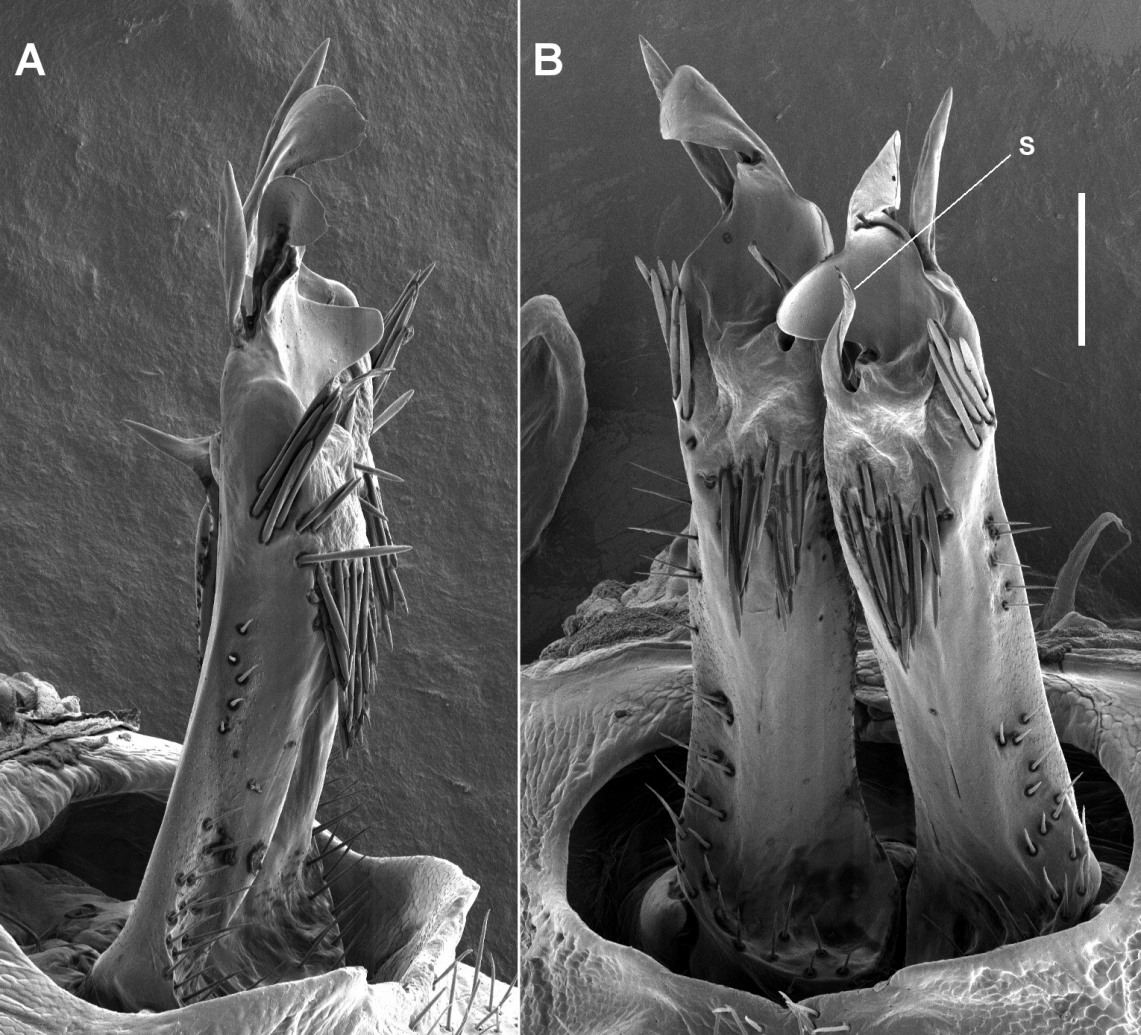
*Tasmaniosoma anubis* sp. n., paratype ex QVM 23:53817; right lateral **A** and posterior **B** views of gonopods in situ. **s** = solenomere. Scale bar = 0.1 mm.

“Male” and “female” in the text refer to adult (stadium 7) individuals. All specimens are stored in 75–80% ethanol in their respective repositories. Gonopods were cleared in 80% lactic acid and temporarily mounted in a 1:1 glycerol:water mixture for examination by optical microscopy. Body measurements were estimated with a Nikon SMZ800 binocular dissecting microscope using an eyepiece scale. Colour photographs were taken with a Canon EOS 1000D digital SLR camera mounted on the same microscope fitted with a beam splitter. The colour images in Figs 1 and 9 are manually stacked composites processed with Zerene Stacker 1.04. Scanning electron microscope images were acquired digitally using a FEI Quanta 600 (Fig. 2) or a Hitachi SU-70 (Figs 3, 6, 8); specimens were examined after air-drying and sputter-coating with platinum. Images and drawings were prepared for publication using GIMP 2.8.

The Suppl. material 1 tabulates data for known specimen lots of all *Tasmaniosoma* species as of 15 February 2015 (data also available online in Mesibov 2006–2015). Locality details are given with latitude and longitude based on the WGS84 datum. My estimate of the uncertainty for each locality is the radius of a circle around the given position, in metres or kilometres.

Abbreviations in text: QVMAG = Queen Victoria Museum and Art Gallery, Launceston, Tasmania, Australia; Tas = Tasmania; WAM = Western Australian Museum, Perth, Western Australia, Australia.

## Results

### Taxonomy Order Polydesmida Pocock, 1887 Suborder Dalodesmidea Hoffman, 1980 Family Dalodesmidae Cook, 1896

#### Tasmaniosoma

Taxon classificationAnimaliaPolydesmidaDalodesmidae

Verhoeff, 1936

Tasmaniosoma Verhoeff 1936: 11. Attems 1940: 442. Jeekel 1971: 355; 1982: 12; 1983: 146; 1984: 85; 1985: 52. Hoffman 1980: 150, 185. Mesibov 2010: 33.

##### Type species.

*Tasmaniosoma armatum* Verhoeff, 1936, by monotypy.

##### Other assigned species.

*Tasmaniosoma alces* Mesibov, 2010, *Tasmaniosoma anubis* sp. n., *Tasmaniosoma aureorivum* Mesibov, 2010, *Tasmaniosoma australe* Mesibov, 2010, *Tasmaniosoma barbatulum* Mesibov, 2010, *Tasmaniosoma bruniense* Mesibov, 2010, *Tasmaniosoma cacofonix* Mesibov, 2010, *Tasmaniosoma clarksonorum* Mesibov, 2010, *Tasmaniosoma compitale* Mesibov, 2010, *Tasmaniosoma decussatum* Mesibov, 2010, *Tasmaniosoma fasciculum* Mesibov, 2010, *Tasmaniosoma fragile* Mesibov, 2010, *Tasmaniosoma gerdiorivum* Mesibov, 2010, *Tasmaniosoma hesperium* Mesibov, 2010, *Tasmaniosoma hickmanorum* Mesibov, 2010, *Tasmaniosoma interfluminum* sp. n., *Tasmaniosoma laccobium* Mesibov, 2010, *Tasmaniosoma maria* Mesibov, 2010, *Tasmaniosoma nicolaus* sp. n., *Tasmaniosoma orientale* Mesibov, 2010, *Tasmaniosoma warra* Mesibov, 2010.

#### Tasmaniosoma anubis sp. n.

Taxon classificationAnimaliaPolydesmidaDalodesmidae

http://zoobank.org/FCFF46F0-6D36-4DC4-A0D9-5E89868D26CC

Figs 1A, D , 2A, D, E , 3

##### Holotype.

Male, Trevallyn Nature Recreation Area, Tas, -41.4417 147.0800 ±25 m (Google Earth), 150 m a.s.l., 21 June 2014, R. Mesibov, QVM 23:53863 (ex QVM 23:53817)

##### Paratypes.

15 males, 28 females, details as for holotype, QVM 23:53817.

##### Other material.

41 males and 51 females (see Suppl. material 1 for details).

##### Diagnosis.

Nominate member of the “*anubis* group” within *Tasmaniosoma* (see Discussion), distinguished from *Tasmaniosoma clarksonorum*, *Tasmaniosoma compitale*, *Tasmaniosoma fasciculum*, *Tasmaniosoma hickmanorum* and *Tasmaniosoma nicolaus* sp. n. by the absence of a distally directed cluster of stout, rod-like setae on the posterior surface of the gonopod telopodite; from *Tasmaniosoma barbatulum* by the presence of a lateral apical process and by the absence of a setal cluster on the anterior telopodite surface; from *Tasmaniosoma fragile* by the absence of a setal cluster on the anterior telopodite surface; and from all other “*anubis* group” members by the presence of an anteromedially directed tab on the anterior telopodite surface.

##### Description.

Male/female approximate measurements: length 10/10 mm, midbody paranota width 1.2/1.1 mm, maximum vertical diameter 1.1/1.1 mm. Live and freshly preserved adults with reddish brown head and antennae; body (Fig. 1A, D) with pale, yellowish brown ground colour, reddish brown anterolateral margins on paranota, reddish brown paramedian spots or short oblique streaks dorsally; legs darkening to light reddish brown distally. Reddish brown colouring fades in alcohol; long-preserved animals more or less uniformly pale yellowish brown, sometimes with a pair of yellowish paramedian spots on each metatergite.

Male with head sparsely setose; antennal sockets slightly impressed, separated by ca 2× socket diameter; antennal groove short and shallow. Antenna slender, slightly clavate, when manipulated reaching back to ring 3; antennomere 6 widest, relative antennomere lengths (2,3,6)>(4,5). Collum from above reniform, convex anteriorly, posterior corner rounded. Tergites 2-4 distinctly narrower than more posterior metatergites; overall ring widths 6>(5,head)>(2,4)>(3,collum); rings 6–15 about same width, 16-18 narrowing. In lateral view, margin of ring 2 tergite slightly lower than margins of collum and ring 3 tergite. Ring 2 ventrally on either side without obvious pit. Ring suture and waist distinct on diplosegments (Fig. 1D), no longitudinal striations on waist; prozonites smooth; metatergites with three transverse rows of ca 12 low, rounded tubercles; posterior metatergal margin slightly emarginate medially. Limbus (Fig. 2E) composed of minute, sharply pointed triangular tabs with jagged, pointed margins. Midbody paranota extend metatergite to ca 1.3× width of prozonite; paranota slightly inflated, marginal groove distinct, anterior corner smoothly convex, posterior corner smoothly convex without projecting posteriorly on any rings, with 1–2 very small tooth-like projections, each bearing small seta; lateral margin very slightly convex, in lateral view slightly oblique (anterior lower) at ca 3/4 ring height. Pore formula 5, 7, 9, 10, 12, 13, 15–18; ozopore small, round, opening dorsolaterally close to margin near posterior corner of paranotum. Spiracles (Fig. 2D) small, round, opening on low, dome-like projections; on diplosegments, projections arise just above and anterior to first leg and about midway between leg bases. Sternites moderately setose with setae longer on anterior rings; sternite wider than long, transverse impression distinct, longitudinal impression indistinct. Anterior legs with prefemur greatly swollen dorsally, femur less so; swellings begin leg 3, decrease gradually to ca leg 15. Midbody legs with tarsus long, slightly curved, ca 1.6× as long as femur; relative podomere lengths tarsus>femur>prefemur>>(postfemur, tibia). Sphaerotrichomes on tarsus and tibia of anterior legs, shafts tapering to point and inclined strongly towards podomere surface. Sparse brush setae on coxa/trochanter, prefemur, base of femur; brush setae unbranched, tapering to blunt point. Pre-anal ring moderately setose; hypoproct subtrapezoidal; epiproct from above tapering smoothly to rounded tip, extending slightly past anal valves. Spinnerets in square array.

Gonopore on distomedial bulge of leg 2 coxa, protected by tall, thin cowl. Short brushes of setae on sternite between legpairs 4 and 5. Legs 6 and 7 bases (Fig. 2A) well- and equally separated; no sternal tab by leg 6; tall, rounded sternal tab by leg 7 with medial brush of thick, rod-like, pointed setae; leg 7 coxa with short, rounded distomedial bulge.

Gonopod aperture ovoid, ca 1/2 as wide as ring 7 prozonite, posterolateral margin raised. Gonocoxa short, subcylindrical, slightly tapering distally. Telopodites (Fig. 3) almost straight, parallel; extending nearly to leg 6 bases when retracted. Telopodite straight, subcylindrical, divided at about 3/4 telopodite height into three major processes: (a) short, slightly helicoid, pointed solenomere posteromedially, directed distally; (b) large, wide, anteroposteriorly flattened central process with apex twisted and flattened mediolaterally with rounded apical margin and narrow bursa-like fold at apex base, and with medial margin of central process curving posteriorly as rounded tab; (c) long, rod-like, tapering, apically rounded lateral process directed distally and slightly laterally. Telopodite projecting posteriorly at base as thin shelf, concave distally. Anterior surface of telopodite at about 2/3 telopodite height with tapering, anteromedially directed tab, longer than wide. Sparse, fine, mainly short setae on lateral and posterolateral surfaces to about 1/2 telopodite height, and on basal shelf. Two closely packed clusters of stout, rod-like, pointed setae: one (ca 12 setae) on posterior telopodite surface, arising just over 1/2 telopodite height and directed basally, the other (ca 10 setae) arising posterolaterally at level of solenomere base and directed distally and slightly posteriorly. Prostatic groove running more or less straight to base of solenomere on medial surface.

Female with legs more slender and prefemora and femora not swollen. Epigynum ca 1/3 width of ring 2, posterior margin produced medially as small, rounded triangle with irregular margin. Cyphopods not examined. (See also Remarks, below.)

##### Distribution.

**Figure 4.**
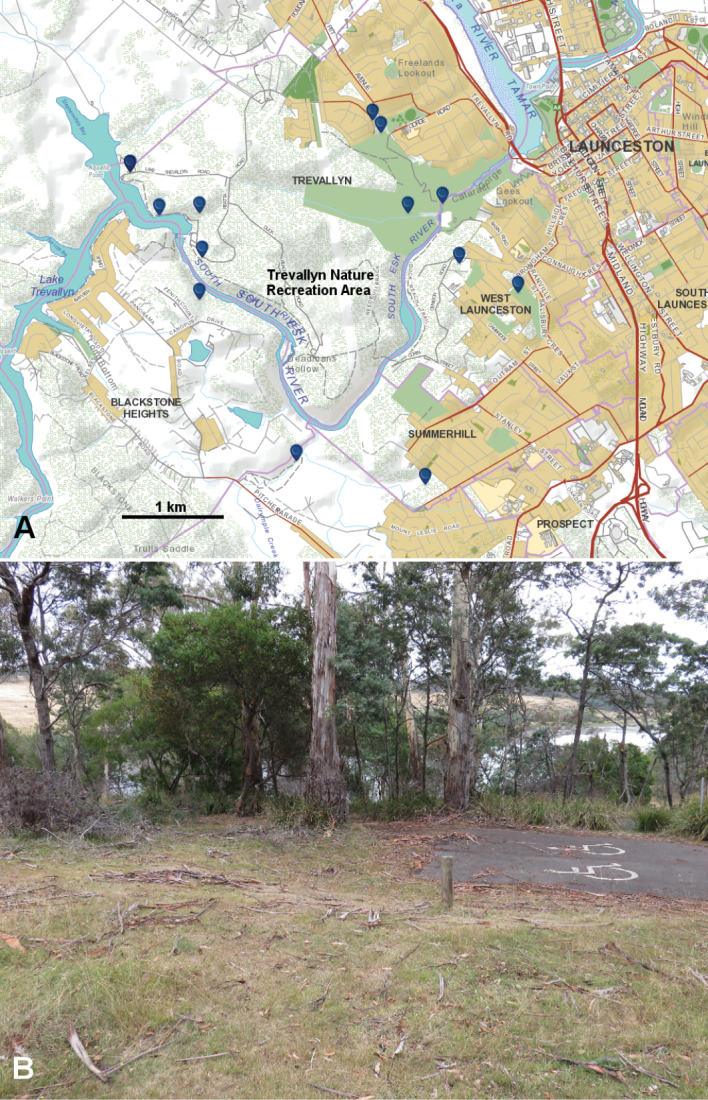
**A** Museum specimen localities to 15 February 2015 of *Tasmaniosoma anubis* sp. n. (blue markers). Base map from http://maps.thelist.tas.gov.au/listmap/app/list/map; for general location see index map, Fig. 5A. **B** *Tasmaniosoma anubis* sp. n. type locality, looking west to Lake Trevallyn. Accumulations of bark litter at the base of *Eucalyptus viminalis* (the tall, white-barked trees at top centre) are preferred shelters for *Tasmaniosoma anubis* sp. n.

**Figure 5.**
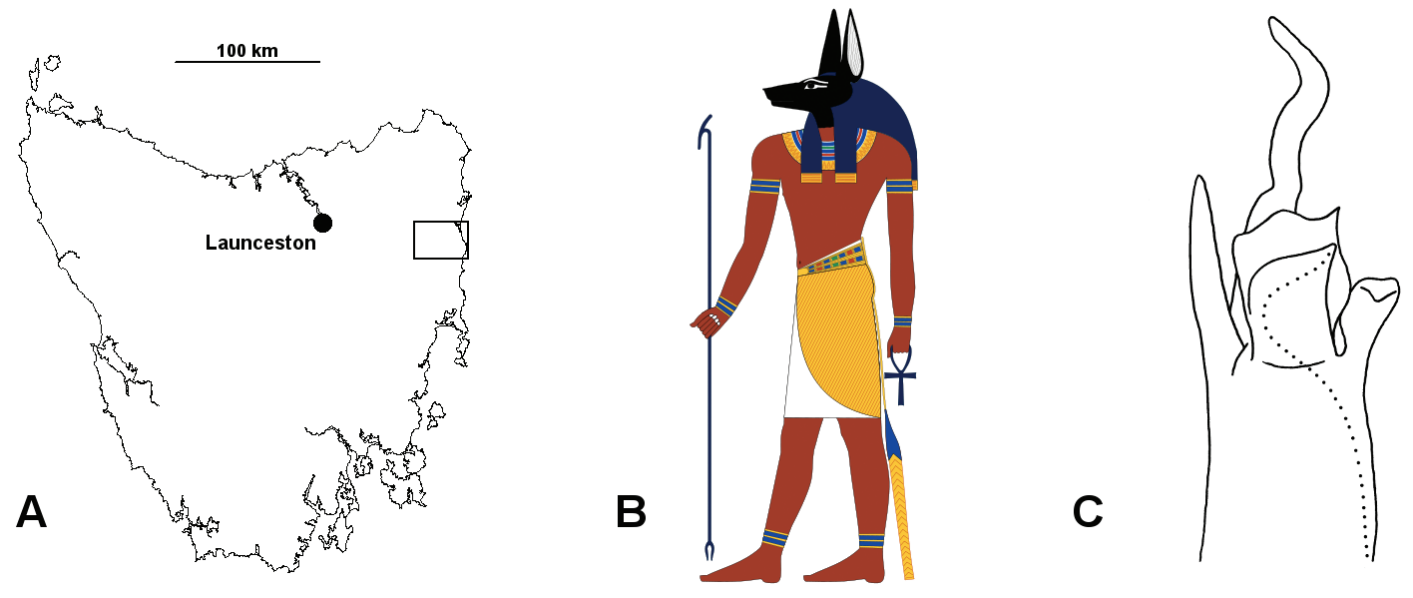
**A** The main island of Tasmania (Mercator projection) showing locations of Launceston (*Tasmaniosoma anubis* sp. n. range) and the Nicholas Range area (rectangle; ranges of *Tasmaniosoma interfluminum* sp. n. and *Tasmaniosoma nicolaus* sp. n.) **B** Anubis, as illustrated by Jeff Dahl (Creative Commons Attribution-ShareAlike license, GNU Free Documentation License; http://commons.wikimedia.org/wiki/File:Anubis_standing.svg) **C** Posterior and slightly ventral view of distal portion of right gonopod of *Tasmaniosoma interfluminum* sp. n., ex QVM 23:52262. Dotted line marks course of prostatic groove.

Eucalypt forest and woodland within a range envelope of <12 km in the city of Launceston, Tasmania, with a core habitat area of <6 km^2^ (Figs 4A, 5A). Locally abundant in the core habitat area and readily found in bark litter at the bases of *Eucalyptus viminalis* trees (Fig. 4B). Co-occurs with *Tasmaniosoma armatum* and the introduced julid millipedes *Ommatoiulus moreleti* (Lucas, 1860) and *Cylindroiulus* spp.

##### Name.

Greek “Anubis”, a jackal-headed god of ancient Egypt; noun in apposition. The tip of the gonopod telopodite in posterolateral view resembles popular representations of the head of Anubis (Fig. 5B). The “snout” of the jackal corresponds to the medial tab of the central process, and the “ears” to the the lateral process and the apical portion of the central process. This name was suggested by collector Lisa Clarkson.

##### Remarks.

The core habitat area for *Tasmaniosoma anubis* sp. n. includes the 440 ha Trevallyn Nature Recreation Area (TNRA; Fig. 4A), which is managed as urban parkland. Large populations of *Tasmaniosoma anubis* sp. n. can be found in grassy woodland adjacent to bitumenised roads in the TNRA.

*Tasmaniosoma anubis* sp. n. has not been found more than ca 1.5 km from the South Esk River near its confluence with the Tamar River at Cataract Gorge in the city of Launceston (Fig. 4A). The land snail *Pasmaditta jungermanniae* (Petterd, 1879), the pseudoscorpion *Neopseudogarypus scutellatus* Morris, 1948 and the spider *Migas plomleyi* Raven & Churchill, 1989 are also believed to be restricted to the Cataract Gorge area, or to the Gorge area and small outlying localities (Fearn 2003). However, one possible male specimen of *Tasmaniosoma anubis* sp. n., examined years ago and unfortunately now lost, and two possible female specimens (QVM 23:52256) have been collected in small, degraded remnants of native vegetation close to the South Esk River in the town of Evandale, ca 15 km southeast of Launceston. I suspect that *Tasmaniosoma anubis* sp. n. was more widespread along the lower South Esk River in pre-European times, i.e. before the early 19th century. Millipede populations would have declined as sheep grazing degraded the local eucalypt woodlands, and would have disappeared over most of the Launceston area as woodlands were cleared for residential development.

Unlike the similar-sized, co-occurring dalodesmid *Atrophotergum pastorale* Mesibov, 2004 and unlike most *Tasmaniosoma* species, *Tasmaniosoma anubis* sp. n. do not usually run away rapidly when disturbed, but remain “crouched” and stationary on the bark, leaf or wood pieces among which the animals are sheltering.

As with *Tasmaniosoma compitale* Mesibov, 2010 and *Tasmaniosoma hickmanorum* Mesibov, 2010 (Mesibov 2010), most *Tasmaniosoma anubis* sp. n. females are missing their second pair of legs and have plugs of sclerotised scar tissue where these legs have broken off.

#### Tasmaniosoma interfluminum sp. n.

Taxon classificationAnimaliaPolydesmidaDalodesmidae

http://zoobank.org/AD1A118D-F8C7-4DD6-A585-BAE4757AC6B5

**Figure 6.**
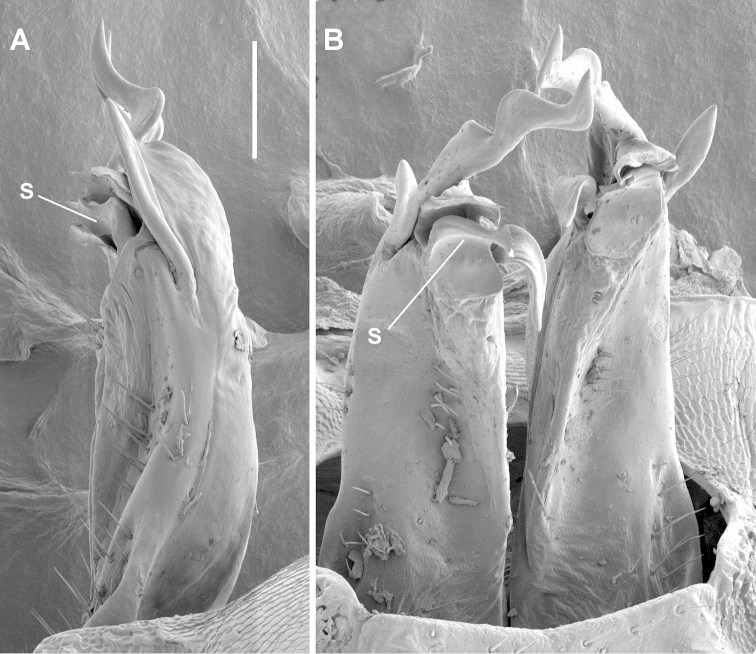
*Tasmaniosoma interfluminum* sp. n., paratype ex QVM 23:52247; left lateral **A** and posterior and slightly ventral **B** views of gonopods in situ. **s** = solenomere. Scale bar = 0.1 mm.

Figs 1B, E , 2B , 5C , 6

##### Holotype.

Nicholas Range, Tas, -41.5417 148.0786 ±25 m (GPS), 570 m a.s.l., 14 May 2012, W. Clarkson and L. Clarkson, QVM 23:53867 (ex QVM 23:52247).

##### Paratypes.

3 males, details as for holotype, QVM 23:52247.

##### Other material.

21 males and 4 females (see Suppl. material 1 for details).

##### Diagnosis.

Very similar to *Tasmaniosoma decussatum*, but without a setose sternal tab adjoining leg 7, with the anterolateral telopodite process prominently notched, with the solenomere tabs curving posteriorly and less deeply separated, and with the medial process tongue-like and curving posterobasally, rather than forming a rounded, mediolaterally flattened, tab-like solenomere extension as in *Tasmaniosoma decussatum*.

##### Description.

Male/female approximate measurements: length 12/11 mm, midbody paranota width 1.4/1.4 mm, maximum vertical diameter 1.1/1.1 mm. Live and freshly preserved adults with yellowish ground colour (Figs 1B, 1E), antennae and distal podomeres pale reddish brown, reddish brown transverse streaks or cellular margins anterior to transverse furrow and near posterior margin of metatergite, and laterally on prozonites and below paranota. Colouring fades in alcohol; long-preserved animals entirely decoloured or more or less uniformly pale yellowish white.

Non-gonopodal features in males mostly as for *Tasmaniosoma anubis* sp. n., but head moderately setose; antennal sockets separated by ca 1.5× socket diameter; overall ring widths 6>5>(4,head)>2>3>collum; rings 8-16 distinctly elongated; ring 2 ventrally on either side with wide, shallow pit, the anterolateral margin well-defined; metatergites with indistinct low tubercles medially; midbody legs with tarsus ca 1.8× as long as femur; brush setae on coxa/trochanter, prefemur and femur of anterior legs; legs 6 and 7 bases (Fig. 2B) well- and equally separated; no setose sternal tab by leg 6 or leg 7, but sternite by leg 7 base slightly produced as rounded bumps.

Gonopod aperture ovoid, ca 1/2 as wide as ring 7 prozonite, posterolateral margin raised. Telopodites (Figs 5C, 6) almost straight, parallel; extending to leg 6 bases when retracted. Telopodite with shallow concavity over most of posterior surface, the margin continuing as posterior margin of posterobasal shelf; anterior surface strongly and broadly ridged. Telopodite divided at ca 2/3-3/4 height into four processes: (a) long, rod-like, tapering, apically rounded lateral process directed distally and slightly posteriorly; (b) large anterolateral process curving posteriorly with distinctive, V-shaped notch on anterior edge, S-shaped distal to notch with acuminate apex directed distally and slightly laterally; (c) posteromedial solenomere developed as parallel, subquadrangular, transverse tabs separated by narrow slit, curving posteriorly; (d) tongue-like medial process curving basally and directed slightly medially. Sparse setae posterolaterally to ca 1/2 telopodite height, with small, separate patch of fine, short setae on posterior surface ca 1/3–1/2 telopodite height. Prostatic groove running up medial surface of telopodite, curving laterally across posterior surface in slit between solenomere tabs, then running along lateral margin of more basal tab to open near medial corner of tab (Figs 5C, 6).

Female about as robust as male but shorter, with posterior rings not elongated; legs more slender and prefemora and femora not swollen. Epigynum ca 1/3 width of ring 2, posterior margin produced medially as low trapezoid with irregular margin. Cyphopods not examined.

##### Distribution.

**Figure 7.**
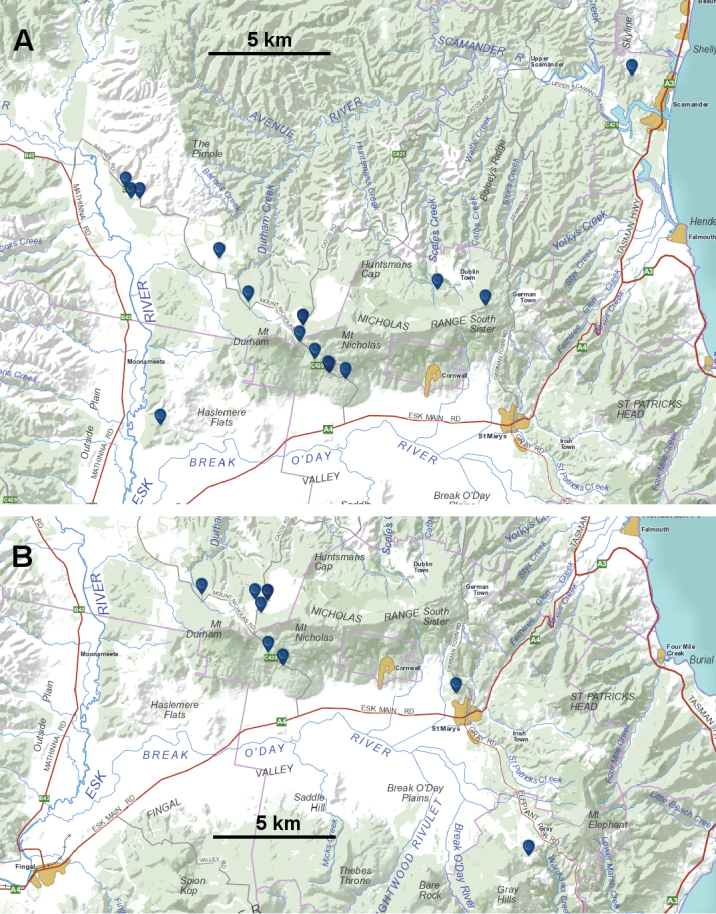
Known localities (blue markers) to 15 February 2015 of *Tasmaniosoma interfluminum* sp. n. (**A**) and *Tasmaniosoma nicolaus* sp. n. (**B**). Base map from http://maps.thelist.tas.gov.au/listmap/app/list/map; for general location see index map, Fig. 5A.

Eucalypt forest over ca 100 km^2^ north and east of Fingal, Tasmania, with an outlying record near the town of Scamander on the east coast (Figs 5A, 7A). Co-occurs with *Tasmaniosoma barbatulum*, *Tasmaniosoma nicolaus* sp. n. and *Tasmaniosoma orientale* in bark and leaf litter in the Nicholas Range.

##### Name.

Latin *inter* (between) + *fluminum* (genitive plural of *flumen* = river); adjective. The largest populations of this species are found between the upper South Esk and Break O’Day Rivers.

##### Remarks.

The gonopod telopodite structure of *Tasmaniosoma interfluminum* sp. n. is remarkably similar to that of *Tasmaniosoma decussatum*, the two differing only in the details noted above in Diagnosis. Another difference is a setose sternal tab adjoining the base of leg 7: present in *Tasmaniosoma decussatum*, absent in *Tasmaniosoma interfluminum* sp. n. I am unable to distinguish the two species in the field.

#### Tasmaniosoma nicolaus sp. n.

Taxon classificationAnimaliaPolydesmidaDalodesmidae

http://zoobank.org/40D169CB-9D35-4A0A-BA36-F4DDF4FD93FA

**Figure 8.**
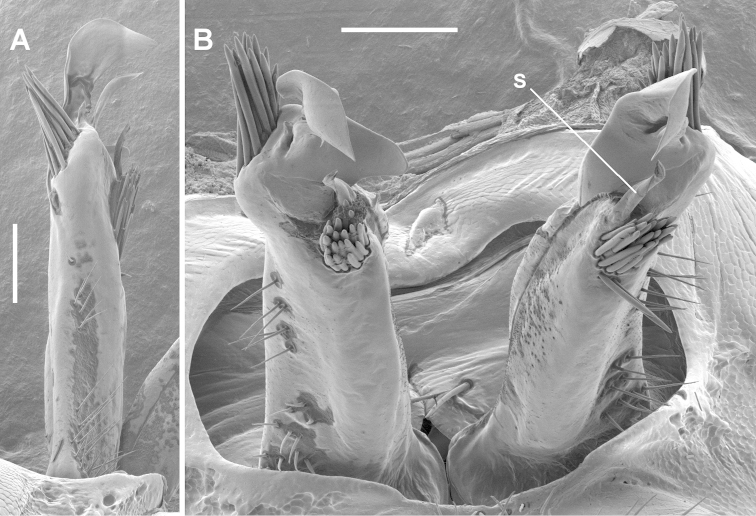
*Tasmaniosoma nicolaus* sp. n., paratype ex QVM 23:53860; lateral view of right gonopod (**A**) and posteroventral view of both gonopods (**B**) in situ. **s** = solenomere. Scale bars = 0.1 mm.

**Figure 9.**
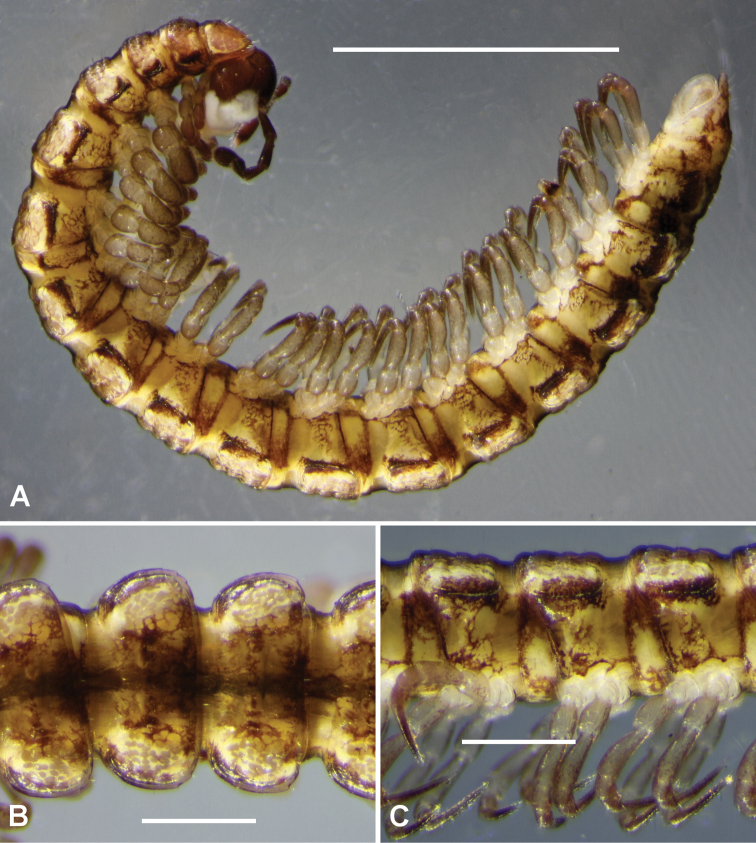
**A** *Tasmaniosoma nicolaus* sp. n., freshly killed male paratype ex QVM 23:53860 **B, C** *Tasmaniosoma barbatulum* Mesibov, 2010, freshly killed male ex QVM 23:53861; dorsal (**B** anterior to left) and right lateral (**C** anterior to right) views of midbody rings. Scale bars: **A** = 2.5 mm, **B, C** = 0.5 mm.

Figs 1C, F , 2C , 8 , 9A

##### Holotype.

Male, Catos Road, Tas, -41.5350 148.0842 ±50 m (GPS), 520 m a.s.l., 9 February 2015, R. Mesibov, QVM 23:53864 (ex QVM 23:53860).

##### Paratypes.

18 males, details as for holotype, QVM 23:53860.

##### Other material.

5 males and 3 females (see Suppl. material 1 for details).

##### Diagnosis.

Member of the “*anubis* group” within *Tasmaniosoma* (see Discussion); distinguished from *Tasmaniosoma anubis* sp. n., *Tasmaniosoma barbatulum* and *Tasmaniosoma fragile* by the absence of a basally directed cluster of stout, rod-like setae on the posterior surface of the gonopod telopodite; from *Tasmaniosoma clarksonorum*, *Tasmaniosoma compitale* and *Tasmaniosoma hickmanorum* by the absence of a lateral process on the telopodite apex; and from *Tasmaniosoma fasciculum* by the telopodite apex extending as a mediolaterally flattened process in the shape of a bird’s head pointed posteriorly.

##### Description.

Male/female approximate measurements: length 11/10 mm, midbody paranota width 1.3/1.2 mm, maximum vertical diameter 1.0/1.0 mm. Live and freshly preserved adults (Figs 1C, F, 9A) with dark reddish brown head and antennae; body with yellowish ground colour, dark reddish brown on paranotal margins, dorsolaterally on prozonites, as cellular borders in patches laterally, and as diffuse streaks and cellular borders dorsomedially and as margin lines posteriorly on pro- and metazonites; legs darkening slightly distally. Long-preserved animals almost entirely decoloured.

Non-gonopodal features in males as for *Tasmaniosoma anubis* sp. n., but overall ring widths 6>5>(4,head)>(3,2)>collum; prefemoral swellings to about leg 20. Legs 6 and 7 bases (Fig. 2C) well- and equally separated; no sternal tab by leg 6; tall, rounded sternal tab by leg 7 with medial brush of thick, rod-like, pointed setae; leg 7 coxa without distomedial bulge.

Telopodites (Fig. 8) almost straight, parallel; extending nearly to leg 6 bases when retracted; without prominent posterobasal shelves. Telopodite straight, subcylindrical, at about 3/4 telopodite height swelling distolaterally, centrally and medially projecting as large anteroposteriorly flattened process with apex twisted to be mediolaterally flattened, shaped like bird’s head and pointed posteriorly; smaller sickle-shaped process arising at base of apex and directed posterodistally; and medial margin of large process curving posteriorly as thin tab with rounded margin. Solenomere short, slightly helicoid, pointed, arising posterior to base of large process and directed distally. Sparse, fine, fairly long setae on lateral and posterolateral surfaces to about 1/2 telopodite height. Two closely packed clusters of stout, rod-like, pointed setae: one (ca 12 setae) on anterolateral surface near base of large telopodite process and directed distally and slightly anteriorly, the other (ca 15 setae) arising posteromedially just posterior to solenomere base and directed distally and slightly posteriorly. Prostatic groove running more or less straight to base of solenomere on medial surface.

Female with legs more slender and prefemora and femora not swollen. Epigynum ca 1/3 width of ring 2, posterior margin produced medially as small, rounded triangle with irregular margin. Cyphopods not examined. (See also Remarks, below.)

##### Distribution.

Eucalypt forest over <40 km^2^ on the Nicholas Range and the Mt Elephant area at the eastern end of the Fingal Valley in northeast Tasmania, on both north- and south-facing slopes at ca 300–500 m a.s.l. (Figs 5A, 7B). Co-occurs with *Tasmaniosoma barbatulum*, *Tasmaniosoma interfluminum* sp. n. and *Tasmaniosoma orientale* in bark and leaf litter.

##### Name.

Latinised “Nicholas” for the Nicholas Range, type locality of this species; noun in apposition.

##### Remarks.

All of the known *Tasmaniosoma nicolaus* sp. n. collection sites are in forest patches with evidence of past logging and burning, and part of the known range of *Tasmaniosoma nicolaus* sp. n. is within the ca 800 ha Nicholas Range Regional Reserve. Like *Tasmaniosoma anubis* sp. n., *Tasmaniosoma nicolaus* sp. n. can be locally abundant: I found most of the 19 type specimens in bark litter under two *Eucalyptus* trees.

My identification of three females as *Tasmaniosoma nicolaus* sp. n. (QVM 23:53635) is tentative. Two of the three females are missing legs 2.

### Note on *Tasmaniosoma barbatulum*

Alcohol-preserved specimens of *Tasmaniosoma barbatulum* are almost entirely decoloured, i.e. more or less uniformly pale white (Mesibov 2010). Live and freshly preserved specimens are coloured very similarly to *Tasmaniosoma nicolaus* sp. n. (Fig. 9). Where the two species co-occur they can be hard to distinguish in the field, but *Tasmaniosoma nicolaus* sp. n. is a little longer and distinctly more robust than *Tasmaniosoma barbatulum*.

## Discussion

The new species *Tasmaniosoma anubis* sp. n. and *Tasmaniosoma nicolaus* sp. n. join *Tasmaniosoma barbatulum*, *Tasmaniosoma clarksonorum*, *Tasmaniosoma compitale*, *Tasmaniosoma fasciculum*, *Tasmaniosoma hickmanorum* and probably *Tasmaniosoma fragile* in a well-defined group within *Tasmaniosoma*, first recognised in Mesibov (2010) and here called “the *anubis* group”. Species in this group have complex but fugitive colouration; three rows of low, rounded, metatergal tubercles; a sternal tab with thick setae at the base of leg 7; and a gonopod telopodite with two or three tight clusters of stout, rod-like setae at or near the telopodite apex. The group occurs over most of Tasmania but has not yet been found in the far south or the southeast of the main island, or on islands in Bass Strait.

The relationships of *Tasmaniosoma interfluminum* sp. n. are uncertain, but it is clearly very close to *Tasmaniosoma decussatum* and the two species may be parapatric in northeast Tasmania.

## Supplementary Material

XML Treatment for Tasmaniosoma

XML Treatment for Tasmaniosoma anubis

XML Treatment for Tasmaniosoma interfluminum

XML Treatment for Tasmaniosoma nicolaus

## Supplementary materials

Supplementary material 1 Specimen records of *Tasmaniosoma* species. Data type: Tab Separated Value File (tsv). Explanation note: Specimen records of *Tasmaniosoma* species as of 15 February 2015. This dataset is made available under the Open Database License (http://opendatacommons.org/licenses/odbl/1.0/). The Open Database License (ODbL) is a license agreement intended to allow users to freely share, modify, and use this Dataset while maintaining this same freedom for others, provided that the original source and author(s) are credited.Robert Mesibov
